# Analysis of the safety and feasibility of sleeve resection under UniVATS after neoadjuvant chemotherapy combined with immunotherapy for locally advanced central-type non-small cell lung cancer

**DOI:** 10.1186/s12957-024-03462-x

**Published:** 2025-03-14

**Authors:** Bo Yang, Li-Wen Zhang, Yu Zhou, Yang-Yun Li, Gui-Dong Shi, Hao Yang, Yue Zhang, Cheng-Cheng Zhang, Mao-Yong Fu

**Affiliations:** Department of Thoracic Surgery, The Affiliated Hospital of North Sichuan Medical College, Nanchong, 637000 Sichuan China

**Keywords:** Non-small cell lung cancer(NSCLC), Sleeve resection, Neoadjuvant chemotherapy

## Abstract

**Objective:**

To investigate the safety and feasibility of sleeve resection under Uni-VATS following neoadjuvant chemotherapy combined with immunotherapy for locally advanced central-type non-small cell lung cancer(NSCLC).

**Methods:**

We analyzed 10 cIIB-IIIB NSCLC patients who underwent sleeve lung resection under single-port thoracoscopy from December 2022 to August 2023 after receiving platinum-based chemotherapy combined with albumin paclitaxel and programmed cell death protein-1 (PD-1) inhibitor drugs. Perioperative clinical data, side effects during neoadjuvant therapy, operation time, intraoperative blood loss, conversion rate to open thoracotomy, postoperative duration of chest tube placement, postoperative drainage volume, postoperative complications, and tumor outcomes were recorded.

**Results:**

This study included 10 patients. The preoperative clinical staging distribution was as follows: Stage IIB, 1 case; Stage IIIA, 5 cases; and Stage IIIB, 4 cases. Imaging evaluation after neoadjuvant therapy revealed that none of the patients achieved complete remission, whereas partial remission and stable disease were observed in 7 cases and 3 cases, respectively. All patients successfully underwent surgery, of which 2 patients required conversion to open thoracotomy (conversion rate, 20%) and 8 patients underwent single-port thoracoscopic minimally invasive surgery. Notably, 2 patients underwent sleeve resection of the right upper lobe, 2 patients underwent sleeve resection of the right middle and lower lobes, 2 patients underwent sleeve resection of the left upper lobe, and 4 patients underwent sleeve resection of the left lower lobe. The average operation time was 236 ± 87.7 min, the average intraoperative blood loss was 168 ± 62.5 mL, the average duration of chest tube placement was 5 days, the average total drainage volume was 1012 ± 464 mL, and the average hospitalization duration was 7 days. One patient developed encapsulated pleural effusion after surgery and underwent computed tomography (CT)-guided puncture drainage. At the 3-month and 6-month follow-up visits, no patient reported any particular discomfort, and chest radiography and CT revealed no abnormalities or signs of tumor recurrence.

**Conclusion:**

Sleeve resection after neoadjuvant chemotherapy combined with immunotherapy for locally advanced central-type NSCLC under single-port thoracoscopy is safe and feasible and provides short-term postoperative benefits for patients.

## Introduction

Lung cancer, a prevalent malignant tumor worldwide, is one of the leading causes of cancer-related mortality [1]. Non-small cell lung cancer (NSCLC) accounts for the majority of lung cancer cases [2]. Recently, with the widespread application of low-dose computed tomography (LDCT) for screening lung cancer cases, a downward trend has been observed in the incidence and mortality rate of lung cancer [3]. However, some NSCLC patients are still diagnosed at an advanced stage [4], with an approximate 5-year survival rate of only 30% [5]. New adjuvant therapy has emerged as an effective option for treating locally advanced NSCLC, as it can downstage the tumor, reduce or eliminate metastatic lesions and lymph nodes, and prolong postoperative survival [6–9].

Currently, evidence from relevant studies has revealed that sleeve lobectomy yields high survival rates in the treatment of central-type NSCLC, without increasing the recurrence rate or postoperative complications. Therefore, it should be considered the preferred approach for central lung cancer [10, 11]. However, after neoadjuvant therapy, tumor regression results in fibrous-fibroblastic necrosis or fibrosis, leading to pulmonary interstitial exudation and mediastinal adhesion, which increases the difficulty of lymph node dissection, exacerbates the damage to elastic fibers in blood vessels, and causes vascular wall degeneration [12, 13]. Consequently, surgery becomes more challenging. With the popularity of video-assisted thoracic surgery (VATS), minimally invasive surgery has become the preferred treatment for NSCLC. Sleeve resection under thoracoscopy has always been a difficult aspect of thoracic surgery.

Uniportal VATS (UniVATS) sleeve resection is associated with several issues, such as different perspectives and instrument interference, which require the surgeon to have high surgical skills. Therefore, whether UniVATS-assisted sleeve resection is a safe and feasible surgical approach for locally advanced central lung cancer after neoadjuvant therapy remains inconclusive.

Undergoing neoadjuvant therapy preoperatively could lead to adverse perioperative outcomes [14]. UniVATS-assisted sleeve resection after neoadjuvant chemotherapy combined with immunotherapy is associated with a high incidence of postoperative complications. Therefore, this study aimed to explore the perioperative complications and short-term efficacy of UniVATS-assisted sleeve resection after neoadjuvant chemotherapy combined with immunotherapy.

## Methods

### Study design and patient selection

This was a retrospective study and written consent was obtained from the patients. Clinical data of patients who underwent sleeve resection after neoadjuvant immunotherapy at the Affiliated Hospital of North Sichuan Medical College from December 2022 to August 2023 were retrospectively collected. Our study included only locally advanced (Stage II-III) NSCLC patients who received neoadjuvant therapy. All patients underwent chest CT to assess the size and location of the tumor. Brain magnetic resonance imaging, abdominal ultrasound, and bone scans were used to determine the presence or absence of distant metastasis. CT-guided percutaneous puncture biopsy or fiberoptic bronchoscopy biopsy was performed for all patients for accurate staging and pathologic analysis. The staging was evaluated before neoadjuvant therapy, after neoadjuvant therapy, and postoperatively according to the 8th edition TNM system of the American Joint Committee on Cancer [15]. This study protocol was approved by the Ethics Committee of the Affiliated Hospital of North Sichuan Medical College (2024ER72-1).

### Neoadjuvant therapy strategy

All neoadjuvant therapy strategies were determined through multidisciplinary team discussions, involving oncologists and thoracic surgeons, with the patient’s informed consent. Neoadjuvant therapy comprised at least 2 cycles of treatment based on platinum-based doublet chemotherapy combined with sintilimab injection, with a treatment interval of 21 days (specific chemotherapy cycles are detailed in Table 1). Each cycle monitored the patient’s adverse reactions and assessed the lesion size through CT evaluation. Notably, the neoadjuvant therapy cycles were not fixed, and before each hospitalization, the thoracic surgeon evaluated whether curative resection of the lesion could be achieved based on the CT findings. However, a minimum of 2 cycles of neoadjuvant therapy were performed.

### Surgical approach and technique

**Fig. 1:**
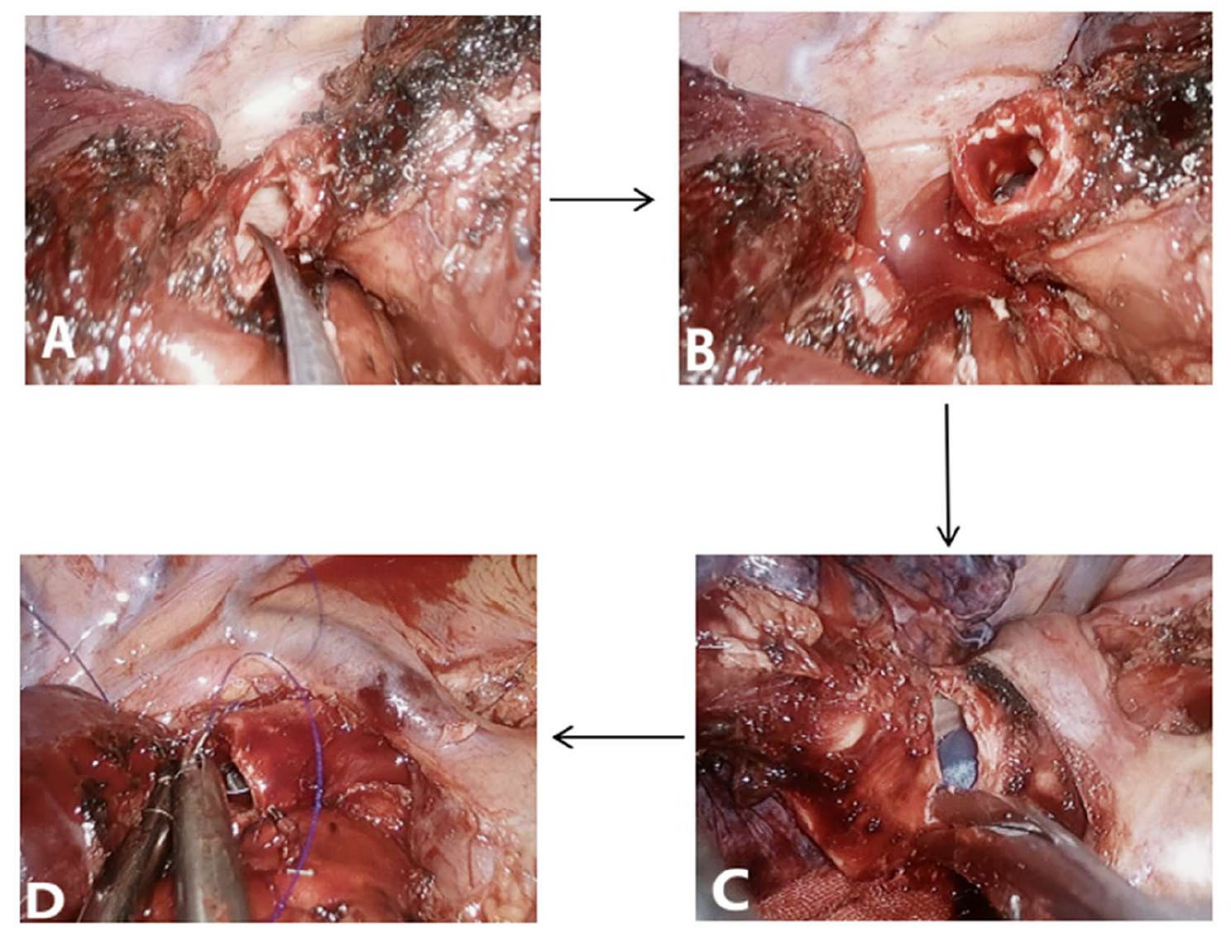
**A**: Cutting the bronchus of the middle segment of the right lung; **B**: After cutting the bronchus of the middle segment of the right lung; **C**: Cutting the right main bronchus; **D**: Anastomosing the cut end of the bronchus

**Fig. 2:**
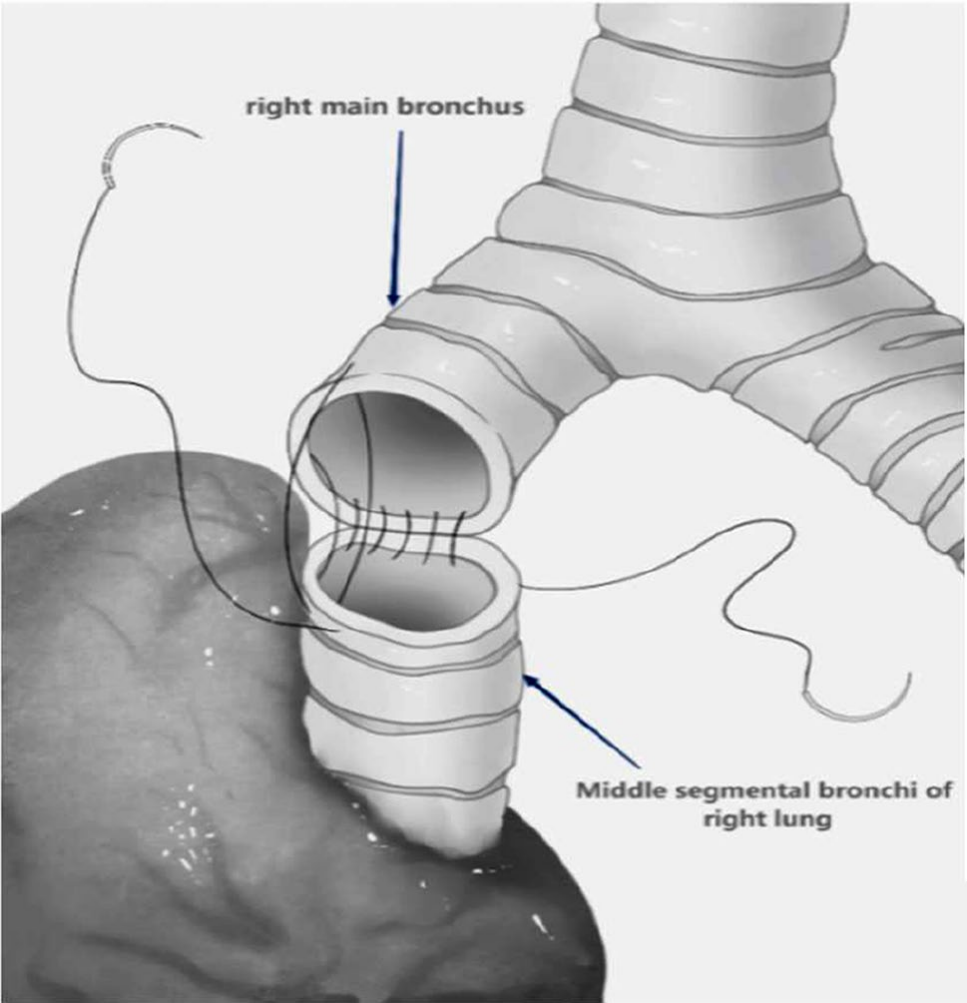
Illustration of right upper lobe sleeve resection

The surgical time for all patients was chosen as 4–6 weeks after the last cycle of neoadjuvant therapy. Sleeve resection via UniVATS was the surgical approach selected for all patients. A surgical incision of 4–5 cm was made at the fifth rib (the fourth rib can be selected when the tumor is located in the upper lobe of the right lung) between the midaxillary line and anterior axillary line. Intraoperatively, the procedure was converted to open surgery if there was a risk of major intra-thoracic bleeding. Sleeve resection was defined as the resection of the affected lobe and a segment of the involved bronchus, followed by anastomosis of the upper and lower bronchial ends, aiming to preserve the adjacent normal lung lobes and avoid complete pneumonectomy on one side. The surgical procedure was performed by a team of thoracic surgeons with extensive experience in sleeve resection. The surgical process included systematic dissection of the pulmonary and mediastinal lymph nodes (right side: stations 2, 4, 7, 8, 9, and 10; left side: stations 4, 5, 6, 7, 8, 9, and 10) and sleeve resection of the bronchus. A frozen section biopsy of the resection margin was performed before bronchial anastomosis to ensure no residual tumor, followed by end-to-end anastomosis. When the tumor invaded the central vessels of the lung, sleeve resection combined with bronchovascular anastomosis or vascular wall reconstruction was chosen. All anastomoses were performed using 3 − 0 Prolene continuous sutures(The surgical process diagram is shown in Fig. 1, The schematic diagram of bronchial sleeve resection is shown in Fig. 2). Two chest tubes were placed at the top and bottom of the pleural cavity postoperatively to facilitate lung re-expansion and drainage. If there was no air leakage and the drainage volume was < 100 mL in 48 h, the chest tubes were removed on the 3rd day postoperatively. Outpatient follow-up was conducted at 1, 3, 6, and 12 months, postoperatively. During the first follow-up visit, chest radiography was performed. Subsequent follow-up visits included serum tumor marker assessment, chest CT, and abdominal and cervical lymph node color Doppler ultrasound examination.

## Results

### General patient characteristics

This study included 10 patients with Stage IIB–IIIB NSCLC. Table 1 shows the baseline characteristics of the patients. All 10 patients were male, with tumor location in the left upper lobe in 2 cases, in the left lower lobe in 4 cases, in the right upper lobe in 2 cases, and in the right middle and lower lobes in 2 cases.

### Neoadjuvant therapy efficacy

**Fig. 3:**
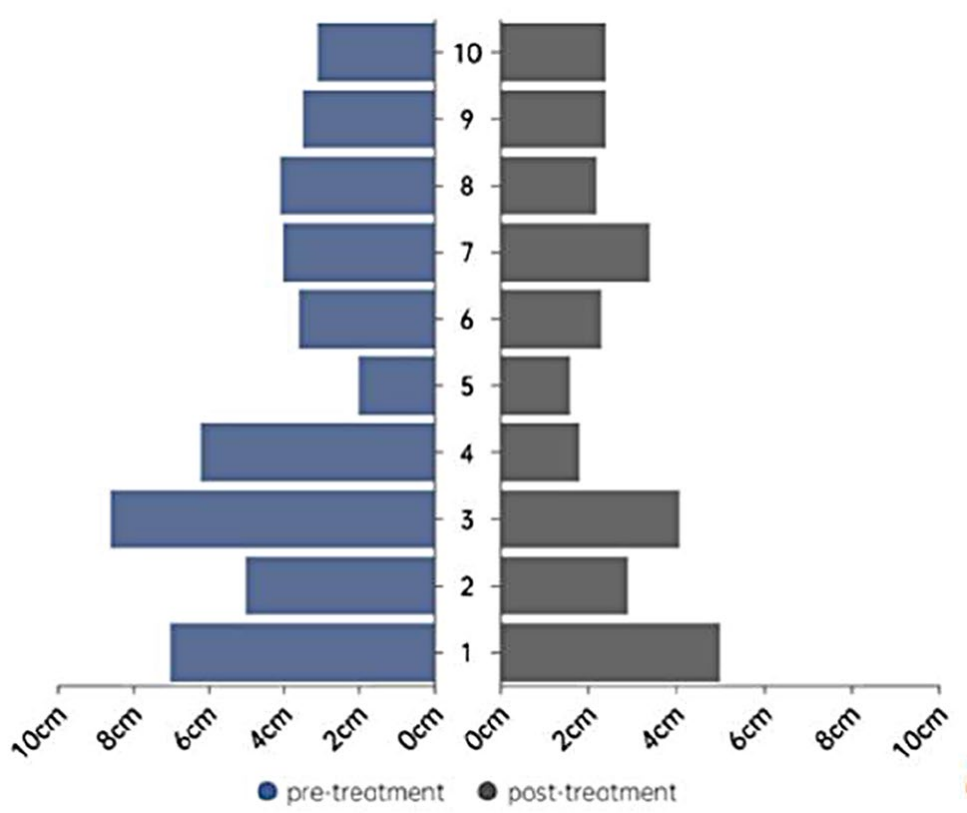
Changes in the tumor diameter before and after treatment in 10 patients

**Fig. 4:**
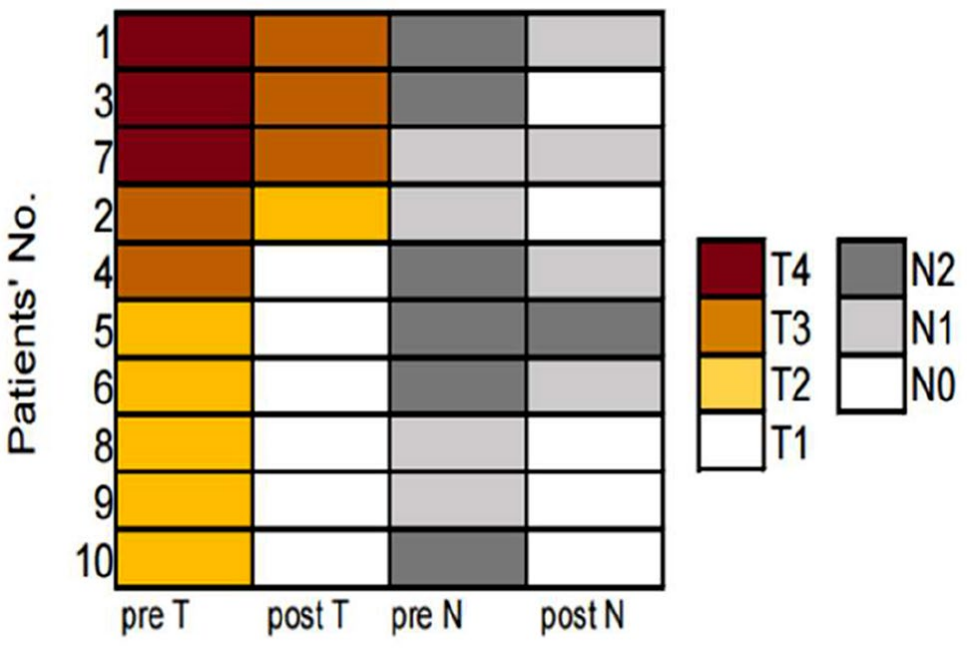
Heatmaps illustrating T and N staging before and after treatment in 10 patients

All patients received at least 2 cycles of platinum-based doublet chemotherapy combined with sintilimab. According to the radiological evaluation after neoadjuvant therapy, none of the patients achieved complete remission. Partial remission was observed in 7 patients (70%), while the remaining 3 patients (30%) had stable disease without disease progression(Figs. 3 and 4).

### Intraoperative and postoperative conditions

**Fig. 5:**
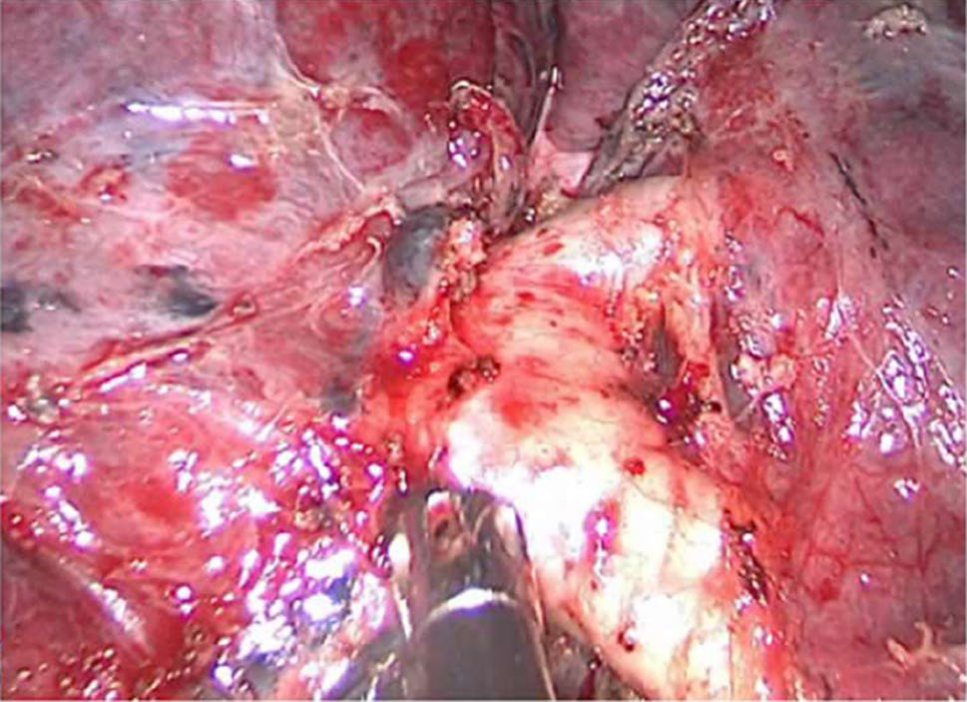
Tumor invasion of the left main pulmonary artery

**Fig. 6:**
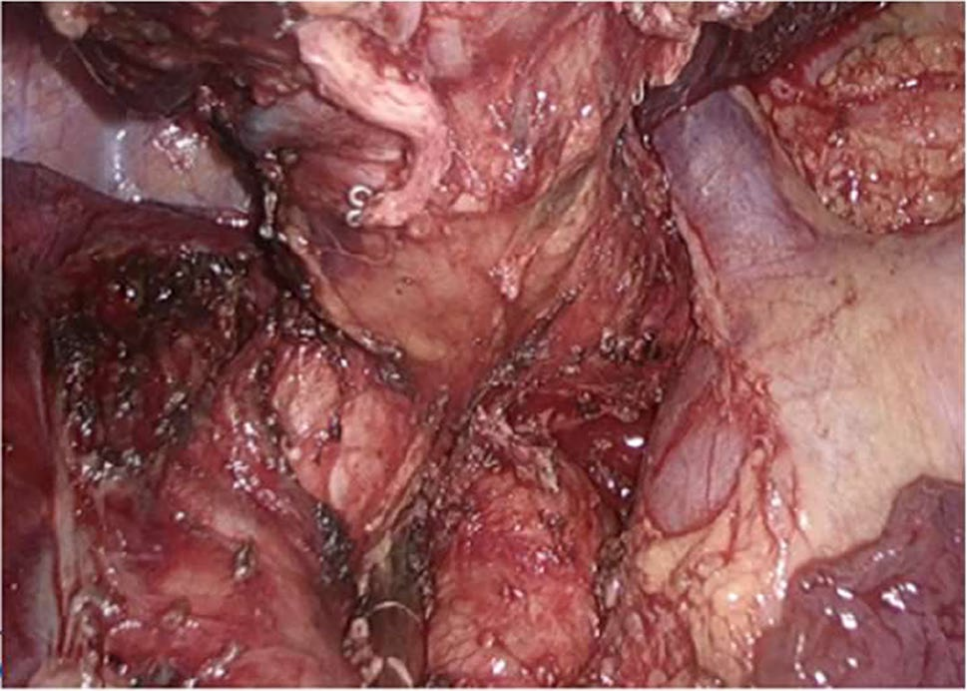
Tumor invasion of the right upper lobe bronchus

All 10 patients underwent surgical treatment. Intraoperatively, dense fibrosis in the pulmonary hilum was detected in 2 patients, leading to conversion to open surgery (conversion rate, 20%). The remaining 8 patients underwent surgery via UniVATS. Involvement of the left pulmonary artery trunk was noted in 2 patients(Fig. 5) while 6 patients showed direct invasion of the bronchus(Fig. 6). Two patients underwent sleeve resection of the right upper lobe, two underwent sleeve resection of the right middle and lower lobes, two underwent sleeve resection of the left upper lobe, and four underwent sleeve resection of the left lower lobe. The average surgical time was 236 ± 87.7 min, the average intraoperative blood loss was 168 ± 62.5 mL, the average duration of postoperative drainage was 5 days, the total drainage volume was 1012 ± 464 mL, and the average hospitalization duration was 7 days. One patient developed encapsulated pleural effusion and underwent CT-guided drainage; however, no serious postoperative complications or deaths were reported. Pathologically, all 10 patients had squamous cell carcinoma. Postoperative pathological examination revealed that 3 patients (30%) achieved major pathological response (MPR), 4 patients (40%) had partial pathological response, and 3 patients (30%) achieved complete pathological response (PCR) (Table 2).

**Table 1:** General clinical data of patients

Patient number	1	2	3	4	5	6	7	8	9	10
Clinical data										
Sex	Male	Male	Male	Male	Male	Male	Male	Male	Male	Male
Age (years)	72	54	68	55	65	57	58	65	67	54
BMI (kg/m^2^)	21.6	26.7	22.3	24.2	24.0	23.2	26.4	23.9	21.3	25.6
Smoking	Yes	Yes	No	Yes	Yes	Yes	Yes	Yes	No	No
Tumor location	LLL	RML + RLL	LLL	RML + RLL	RUL	LLL	LUL	LLL	RUL	LUL
Adjuvant therapy cycles	3	2	4	3	4	3	4	2	3	3
Operating time (min)	238	210	150	290	350	365	390	130	267	240
Intraoperative bleeding (mL)	170	210	157	325	210	200	115	95	100	180

*BMI* body mass index, *LLL* lower left lung, *RML* middle lobe of right lung, *RLL* right lower lung, *RUL* upper lobe of right lung, *LUL* upper left lung

**Table 2:** Postoperative clinical data of patients

Patient number	1	2	3	4	5	6	7	8	9	10
Clinical data										
Postoperative hospitalization duration (days)	5	4	6	12	6	7	7	6	10	6
Drainage time (days)	4	3	4	10	5	5	6	5	8	5
Postoperative drainage (mL)	752	400	1115	1735	1220	895	1050	1000	1540	810
Complications	No	No	No	Yes*	No	No	No	No	No	No
Tumor results	PPR	PPR	MPR	PCR	PCR	PPR	PPR	MPR	MPR	PCR

*PPR* partial pathological remission, *MPR* major pathological remission, *PCR* complete pathological remission, *: enveloped pleural effusion

## Discussion

In this study, all patients who underwent UniVATS sleeve lobectomy after neoadjuvant chemotherapy or immunotherapy achieved successful surgical treatment with R0 resection. However, 2 cases required conversion to open thoracotomy due to dense adhesions in the pulmonary hilum, which posed a risk of intraoperative bleeding. No severe complications or deaths were reported for any patient postoperatively, except for 1 patient who required CT-guided drainage of encapsulated pleural effusion. In the early 21st century, Santambrogio reported the first case of VATS sleeve lobectomy [16], and this surgical approach has gradually been applied in the treatment of locally advanced central-type NSCLC patients. A meta-analysis of VATS sleeve lobectomy for centrally located NSCLC [17], which included 5 retrospective cohort studies with a total of 436 patients, showed that VATS sleeve lobectomy significantly reduced blood loss, resulted in reduced incidence of surgical trauma, and improved postoperative recovery without compromising the tumor prognosis compared to open sleeve lobectomy. Neoadjuvant chemotherapy or immunotherapy has been studied in operable and locally advanced lung cancer patients and has been shown to downstage tumors and prolong survival []. Currently, immune checkpoint inhibitors (including drugs targeting programmed cell death protein-1 [PD-1] or PD-L1 molecules) are a popular research topic. However, there is a lack of large-sample studies to validate the safety of UniVATS sleeve lobectomy after neoadjuvant chemotherapy or immunotherapy and assess the postoperative benefits for patients. The study by Ling et al. [19]. investigated 31 NSCLC patients who received neoadjuvant anti-PD-1 therapy, with 48.4% of patients achieving MPR or PCR. Zhang et al. [20]. retrospectively analyzed NSCLC patients who received neoadjuvant chemotherapy plus PD-1 inhibitors (PD-1 + chemotherapy group) compared to those who received neoadjuvant chemotherapy alone (chemotherapy group). The statistical analysis showed that the MPR and 2-year DFS rates were higher in the PD-1 + chemotherapy group than in the chemotherapy group (49.3% vs. 19.0% and 79.3% vs. 60.2%, respectively). Another study [21] demonstrated that MPR was associated with high 2-year DFS and overall survival (OS) rates.

Recently, there have been a few studies on the safety of sleeve resection after neoadjuvant therapy. A retrospective study [22] compared the VATS group and the open surgery group, indicating that sleeve resection after neoadjuvant chemoradiotherapy is feasible. Additionally, the perioperative outcomes of both groups were comparable. Another study by Liang et al. [13]. retrospectively included patients who received neoadjuvant chemotherapy combined with immunotherapy (neoadjuvant IO + C group) and patients who received chemotherapy alone (chemotherapy group). The results showed that the MPR rate was higher in the neoadjuvant IO + C group than in the chemotherapy group (50% vs. 10%). However, the neoadjuvant IO + C patients experienced increased surgical difficulty, such as greater disruption of elastic fibers in blood vessels, vascular wall degeneration, fibroblast-like necrosis, and fibrosis. This suggests that although VATS sleeve resection is safe and feasible, neoadjuvant therapy can increase the surgical complexity.

In our study, 2 patients required intraoperative conversion to open surgery due to dense fibrous scar tissue surrounding the pulmonary hilum after tumor regression, which made the separation process extremely challenging. Current research has demonstrated the benefits of neoadjuvant therapy in advanced NSCLC patients. During preoperative imaging evaluation, we observed tumor or lymph node shrinkage, which were favorable conditions for surgery. However, in real clinical practice, we found that corresponding areas after tumor regression would form dense fibrous scars, making it difficult to separate blood vessels and bronchi. Additionally, we observed severe edema in normal tissues of patients who underwent neoadjuvant therapy, which increased vascular fragility. Furthermore, there were 2 cases wherein the lymph nodes that were enlarged preoperatively shrank postoperatively; however, during surgery, these lymph nodes became extremely hardened, posing significant challenges during dissection. These factors collectively contribute to increased surgical difficulty, prolonged operative time, and increased risk of postoperative complications. However, no major complications occurred in our cohort, and the median operative time and blood loss were similar to those reported in previous related studies. Nevertheless, the presence of dense fibrous scars, adhesions, and lymph node sclerosis during surgery increases the complexity of the procedure. Short-term follow-up at 3, 6, 9, and 12 months postoperatively did not reveal any tumor recurrence or surgery-related complications.

This study has several limitations. First, the sample size was small, and the retrospective nature of the study may have introduced a selection bias. Regrettably, our study solely relied on CT imaging to assess the staging of tumors before and after treatment, which may have led to imprecise preoperative staging. In future studies, we aim to incorporate additional examinations such as PET-CT and EBUS to enhance preoperative evaluations. Furthermore, there was no control group, and larger prospective randomized controlled trials are warranted to validate the practicality of these findings. Second, long-term prognostic outcomes were not assessed, and future studies should have longer follow-up periods to observe the 3-year and 5-year DFS and OS rates.

## Conclusion

This study investigated the surgical complications and short-term therapeutic efficacy of sleeve resection for locally advanced NSCLC after neoadjuvant therapy performed using UniVATS. Our findings suggest that single-port thoracoscopic sleeve resection for locally advanced central-type NSCLC after neoadjuvant therapy is feasible, with no occurrence of severe perioperative complications.

## Data Availability

No datasets were generated or analysed during the current study.
